# The Mediating Effects of Work–Life Balance (WLB) and Ease of Using WLB Programs in the Relationship between WLB Organizational Culture and Turnover Intention

**DOI:** 10.3390/ijerph19063482

**Published:** 2022-03-15

**Authors:** Han-Sun Yu, Eun-Jun Lee, Tae-Kyun Na

**Affiliations:** 1 Culinary Team, Holiday Inn Incheon Songdo, Incheon 22008, Korea; gkstjs0217@naver.com; 2 Department of Hotel Cuisine & Restaurant Management, Chungwoon University, Hongsung 32244, Korea; 3 Division of Culinary, Pastry & Baking Arts, Doowon University of Technology, Paju 10838, Korea

**Keywords:** culinary staff, work–life balance, organizational culture, turnover intention, WLB program

## Abstract

Work–life balance (WLB) is an important concern for all workers irrespective of their age, sex, education level, family structure, or occupation. This study analyzes WLB’s mediating effects and the ease of using WLB programs in the relationship between WLB organizational culture of hotels and turnover intention of its culinary staff. We conducted a survey featuring 320 culinary staff members at hotels in Incheon from 10 to 30 August 2020 and performed statistical analysis using 290 responses. We find that the company’s willingness for WLB, empathetic communication with colleagues, material support of colleagues for WLB, and the ease of using WLB programs in organizational culture had a positive impact on WLB. The company’s willingness for WLB, boss’s consideration for WLB, empathetic communication with colleagues, and material support of colleagues for WLB in organizational culture had a negative impact on turnover intention. The ease of using WLB programs had no indirect effect on the relationship between organizational culture and turnover intention. However, WLB had an indirect effect on the relationship between the four components except for the boss’s consideration for WLB and turnover intention. Hotel management should create an organizational culture that supports the WLB of culinary staff.

## 1. Introduction

Work–life balance (WLB) is an important concern for all workers irrespective of their age, sex, education level, family structure, or occupation [1]. Korean workers, who have been working 52 h a week since February 2018, are increasingly seeking to improve their quality of life while maintaining a WLB that helps them take some time off from the work-centric social environment [2]. According to the Ministry of Employment and Labor’s “A Survey on Public Perception of the 52-h workweek,” seven out of ten employees would prefer to enjoy leisure time instead of receiving more wages, promotions, or other economic compensation through overtime [3]. In other words, the old perception that company and work were valued more than personal life and family has been replaced by one that places more value on personal life. However, the average annual working hours of Korean workers is 1908 h, which is higher than the Organization for Economic Cooperation and Development (OECD) average of 1687 h, and Korea’s WLB index ranks 37th among 40 OECD countries. Moreover, Koreans’ life satisfaction (on a scale of 10) is 5.9 points, which is lower than the OECD average of 6.5 points [4]. 

Particularly, culinary staff in hotels, whose social perception and job expectations have recently changed positively as a result of food-related TV programs in Korea [5,6], are not performing to their full potential owing to poor working conditions such as low wages, irregular holidays, unguaranteed breaks, weekend work, excess workload, and overtime hours [7,8]. This working environment not only creates physical and mental health problems by causing a conflict between work and life but also increases the turnover rate of culinary staff. However, hospitality companies with a high reliance on human resources in their service processes often emphasize improving productivity and organizational performance to remain competitive amid rapid business environment changes [9], while paying relatively less attention to WLB, job satisfaction, and career development [10]. Furthermore, millions of employees worldwide have been affected by the global lockdown caused by the COVID-19 pandemic. The Korean government’s social distancing policy to prevent the spread of this epidemic not only changed consumer behavior but also led to a sharp decline in sales owing to a decrease in the number of customers at restaurants [11,12,13]. Consequently, culinary staff members are taking unpaid leave or being laid off. An organization that cares for the needs and well-being of employees will have employees that are self-motivated, satisfied with their work, and comfortable in their work environment [14]. In this context, Cain et al. [11] emphasized that the position of an executive chef is exceptionally important and unique for a restaurant’s success and argued that when the chef achieves WLB, it is ultimately beneficial for the organization. Therefore, currently, when the job instability of culinary staff is greater than ever, it is imperative to improve the WLB of employees to reduce the turnover of hotel culinary staff and retain skilled chefs.

Numerous studies [15,16,17] in the hospitality industry use WLB as a preceding variable to analyze its impact on job satisfaction, job commitment, and turnover intentions. In general, WLB enhances job satisfaction, quality of life, organizational commitment, and work consciousness, while lowering turnover intentions [17,18,19]. Although it is important to understand the effectiveness of WLB, it is equally crucial to identify the antecedent variables that increase WLB in the hospitality industry from a human resource management perspective. Several studies [20,21] have analyzed the influence of managers’ support, work schedule demands, WLB organizational culture, and inefficient work culture on WLB. Recent studies [2,22,23] on general office workers and hospital employees emphasize the importance of organizational culture in enhancing WLB. 

However, most of the previous studies [24,25] have categorized organizational culture into organizational hierarchical culture, developmental culture, consensual culture, and rational culture based on the Computing Values Model and analyzed their effectiveness. There is insufficient research on the effectiveness of WLB organizational culture in the hospitality industry, such as whether companies value employees’ WLB, whether WLB-related programs are easily implemented, and whether bosses are interested in employees’ families, career growth, and leisure time. Therefore, it is necessary to analyze the impact relationship between WLB organizational culture, WLB, and turnover intention among hospitality industry employees, since demand for WLB is growing. Additionally, recent studies in Korea have evaluated WLB organizational culture based on Park and Sohn [26]; however, they do not explore the relationship between the five components developed in this study. Therefore, this study aims to extend the research model by analyzing the effects of the remaining four factors on the ease of using WLB programs among other factors constituting WLB organizational culture. 

Thus, the purpose of this study can be summarized as follows: First, the study aims to analyze the influence relationship between the constituent factors of WLB organizational culture. Second, it analyzes the effect of organizational culture of hotels on the WLB and turnover intention of culinary staff. As a result, this study offers useful implications for enhancing the organizational culture of hotels to promote WLB, raising the level of WLB, and lowering the turnover intentions of culinary staff members.

## 2. Theoretical Background

### 2.1. WLB Organizational Culture

WLB organizational culture is a combination of WLB and organizational culture and is referred to by each researcher differently, such as work–family culture or family-friendly organizational culture. Recently, as the concept of work and family has expanded to encompass all workers, regardless of sex or marital status [27,28], and since life has expanded beyond the existing family problem-centered perspective to include personal leisure, health, and development [29], the term “work and family” or “family-friendly” naturally morphs into the term “work and life” [26,30]. In this context, Kim and Park [29] defined WLB as a perceived balance between work and non-work areas, such as family, leisure, individual growth, and self-development. Thompson et al. [31] defined WLB organizational culture as the shared assumptions, beliefs, and values regarding the extent to which an organization values and supports the integration of employees’ work and life. Nitzche et al. [32] defined WLB organizational culture as helping companies contribute to each member’s personal life. 

As the importance of such a balanced organizational culture between work and life has increased, what it entails and how to measure it has been discussed. Based on previous studies, Thompson et al. [31] categorized WLB organizational culture measures such as the degree to which bosses and organizations support their employees’ family life (managerial support factor), the awareness of career consequences when using such programs (career consequences), and the organization’s demand for prioritizing work over home (organizational time demands). Kim and Kim [33] classified the factors that contribute to a family-friendly corporate culture into three categories: the utilization of a family-friendly system, the organizational culture, and the managers’ support. These studies have limitations, since they arbitrarily and subjectively construct measurement factors to assess WLB organizational culture [26]. To measure WLB organizational culture, Park and Sohn [26] developed a measurement tool composed of five factors, such as the company’s willingness for WLB, boss’s consideration for WLB, empathetic communication with colleagues, material support of colleagues for WLB, and ease of using WLB programs. 

Most of the studies [2,34,35] based on Park and Sohn [26] have analyzed the effects of the five factors constituting WLB organizational culture on WLB, job satisfaction, and life satisfaction. However, it is important to analyze the relationships among these five factors as well as employee attitudes. Numerous studies have found that among the five factors, the other four factors have an impact on the ease of using WLB programs; Lee et al. [36] found that informal support from bosses and colleagues had a positive impact on the ease of using a family-friendly system. According to Woo and Kwak [37], the greater the overall perception of organizational support for families, the higher the intention of employees to use the paternity leave policy. In contrast, Lee and Lee [38] found that an organizational culture that makes it easy for colleagues to coordinate before taking a leave of absence increased the tendency to not utilize leave, while an organizational culture that guarantees leave autonomy did not significantly affect employees’ tendency to not utilize leave. Korean employees are not encouraged to apply for leave, even if an organization creates a family-friendly environment through a reciprocal relationship among its employees. Based on the results of these previous studies, this study established the following hypotheses to determine which of the four WLB organizational culture factors could improve the ease of using WLB programs.

**Hypothesis** **1:** *A boss’s consideration for WLB has a positive impact on the ease of using WLB programs*.

**Hypothesis** **2:** *A company’s willingness for WLB has a positive impact on the ease of using WLB programs*.

**Hypothesis** **3:** *Empathetic communication with colleagues has a positive impact on the ease of using WLB programs*.

**Hypothesis** **4:** *Material support of colleagues for WLB has a positive impact on the ease of using WLB programs*.

### 2.2. WLB Organizational Culture and WLB

In the following section, we present a review of previous studies that have examined the relationship between WLB organizational culture and WLB. Choi and Kim [39] found that among WLB organizational cultures, the ease of using WLB programs has a positive impact on work–family balance and work–leisure balance, and colleagues’ support for WLB also improves work–leisure balance. Conversely, Brown et al. [22] found that many women did not believe that WLB could be attained through WLB programs and therefore did not use such programs. According to Park et al. [40], support from family, as well as colleagues and bosses had a positive impact on working women’s WLB. Sohn and Park [20] identified individual variables (WLB beliefs, resilience), family variables (work–life support, household sharing satisfaction), and organizational variables (WLB organizational culture and inefficient work culture) as factors affecting WLB. Additionally, the self-regulation ability of resilience variables, satisfaction with the household division of family variables, WLB organizational culture of organizational variables, and inefficient work culture have a positive impact on WLB. 

Furthermore, the following representative studies have been conducted on employees in the hospitality industry. Based on previous research, Lee and Han [41] selected organizational sponsorship awareness, job autonomy, and family support as leading variables affecting the WLB of hotel employees. According to the analysis, dual organizational sponsorship awareness and job autonomy had a positive impact on WLB. A study conducted by Na [34] on culinary staff at a luxury hotel in Seoul found that among WLB organizational cultures, a company’s willingness for WLB, a boss’s consideration for WLB, material support of colleagues for WLB, and ease of using WLB programs had a significant positive impact on the WLB of culinary staff members. However, empathetic communication with colleagues did not have a statistically significant impact on WLB. Choi [42] indicated that the exchange relationship between the boss–employee had a positive impact on the WLB of hotel employees. Based on the results of these studies, we established the following hypothesis to analyze the effectiveness of the WLB organizational culture of luxury hotels located in Incheon.

**Hypothesis** **5:** *The WLB organizational culture of a hotel has a positive impact on the WLB of its culinary staff.*

**Hypothesis** **5a:** *A boss’s consideration for WLB will have a positive impact on the WLB of its culinary staff*.

**Hypothesis** **5b:** *A company’s willingness for WLB will have a positive impact on the WLB of its culinary staff*.

**Hypothesis** **5c:** *The ease of using WLB programs will have a positive impact on the WLB of its culinary staff*.

**Hypothesis** **5d:** *Empathetic communication with colleagues will have a positive impact on the WLB of its culinary staff*.

**Hypothesis** **5e:** *The material support of colleagues for WLB will have a positive impact on the WLB of its culinary staff*.

### 2.3. WLB Organizational Culture and Turnover Intention

Turnover intention refers to the intention of a member to leave an organization within a short period of time [43]. Hotel industry employees in Korea have high turnover intentions to transfer to other hotels or industries, although their turnover intentions vary by department, sex, and age, among others [44]. An increasing turnover rate could cause the company to encounter problems such as increased costs for hiring replacements, reduced production capacity during new employees’ training period, loss of experienced employees, and being slandered [45]. In particular, it is important to understand the difference in values between generations of employees to understand the turnover intention of hospitality industry employees [46]. According to research by Brown et al. [22] conducting a survey targeting the Generation Y employees in the hospitality industry, the work–family balance was the main reason for leaving the hospitality industry.

Previous studies have analyzed the relationship between the sub-factors of WLB organizational culture and turnover intentions. First, effective leadership is essential for organizational competitiveness. Mrusek et al. [47] argued that Michelin-starred restaurants with sustainable chefs ensure employee satisfaction, thereby lowering their employee turnover. A study by Abdien [48], which examined employees of a 5-star hotel chain in Egypt, found that the higher the manager’s support and communication among colleagues as well as between employees and supervisors, the lower the turnover intention. According to Lee et al. [49], a supervisor’s empathy, defined as sharing the same feelings, opinions, and arguments as the employees of the organization, lowers the turnover intention of hotel employees. Kim and Chung [50] found that the impersonal supervision of the boss leads to an increase in the turnover intention of culinary staff. A study by Na and Kim [51] targeting culinary staff at luxury hotels found that interpersonal deviant behavior, such as gossiping or rude behavior toward colleagues, increases the cynicism that does not care what happens to bosses, colleagues, and subordinates, and this in turn increases turnover intention. Additionally, Kim and Kim [52] revealed that the hotel employees’ perception of colleague sponsorship reduces their intention to change teams. 

Prior studies have examined the company’s willingness for WLB and the ease of using WLB programs as the sub-factors of WLB organizational culture. Studying full-time academics working in higher education institutions in South India, Devadhasan et al. [53] found that WLB practices reduce academic turnover intentions. Kim et al. [54] found that a family-friendly company culture has a positive effect on the awareness of the family-friendly system, which in turn increases job satisfaction and organizational commitment and decreases turnover intentions. According to Lee et al. [36], the ease with which a family-friendly program can be used has a positive effect on the psychological well-being of workers in the field of arts and culture. Thakur and Bhatnagar [55] found that the current utilization of WLB practices increases intention to stay, and job embedding fully mediates the relationship between the two variables. Based on the results of these studies, our study established the following hypothesis to analyze how the sub-factors of WLB organizational culture affect the turnover intention of employees of luxury hotels located in Incheon.

**Hypothesis** **6:** *The WLB organizational culture of a hotel has a negative impact on the turnover intention of its culinary staff*.

**Hypothesis** **6a:** *A boss’s consideration for WLB will have a positive impact on the turnover intention of its culinary staff.*

**Hypothesis** **6b:** *A company’s willingness for WLB will have a positive impact on the turnover intention of its culinary staff*.

**Hypothesis** **6c:** *The ease of using WLB programs will have a positive impact on the turnover intention of its culinary staff.*

**Hypothesis** **6d:** *Empathetic communication with colleagues will have a positive impact on the turnover intention of its culinary staff*.

**Hypothesis** **6e:** *The material support of colleagues for WLB will have a positive impact on the turnover intention of its culinary staff*.

### 2.4. WLB and Turnover Intention

Previous studies have analyzed the relationship between WLB and turnover intentions for hospitality industry employees. According to a study by Hong et al. [56], which analyzed the effectiveness of WLB for airline cabin crew members, the overall evaluation of WLB and work–family balance among WLB factors is associated with improved job performance and reduced turnover intentions. Furthermore, Song et al. [57] found that WLB increases job satisfaction, which in turn decreases the turnover intention of employees of low-cost carriers. According to Kang et al. [58], who focused on hotel employees working at the front desk (rewards desk and concierge) and the back-end (kitchen and stewarding), the quality of work and life of employees in the hospitality industry is affected by the service climate and physiological capital, and it reduces turnover intentions. A study by Kaya and Karatepe [59], which surveyed hotel employees in Turkey, found that WLB lowers the propensity to leave early and arrive to work late. Moreover, in a study focusing on hotel employees in Daejeon, Park [15] found that work–growth balance, work–leisure balance, and work–family balance, which are sub-factors of WLB, are all associated with reducing turnover intentions. Particularly, work–growth balance lowers turnover intention the most. Based on the results of these studies, our study established the following hypothesis to analyze the effects of WLB on the turnover intention of culinary staff.

**Hypothesis** **7:** *The WLB of culinary staff has a negative impact on their turnover intention*.

## 3. Materials and Methods

### 3.1. Measurement Model

**Figure 1:**
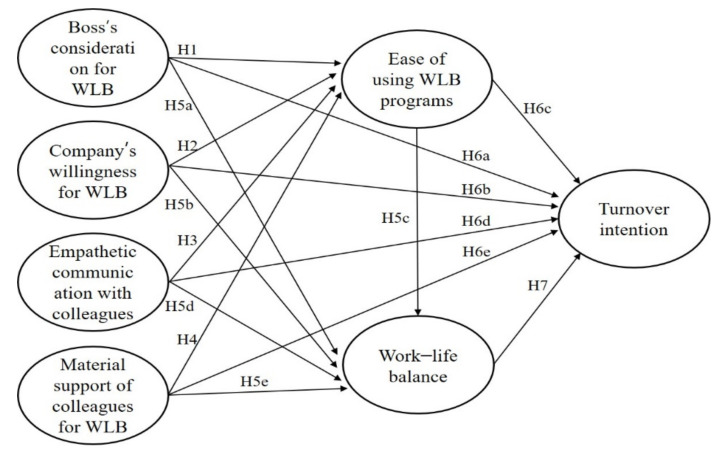
Research model. Note: WLB = work–life balance.

This study sought to analyze the relationship between the five factors constituting WLB organizational culture, as well as the effect of WLB organizational culture on WLB and turnover intention of culinary staff. Hence, based on previous research, it was hypothesized that the ease of using WLB programs would be affected by the remaining four factors, and that the five dimensions of WLB organizational culture would affect WLB and turnover intentions. Figure 1 illustrates our research model. 

### 3.2. Research Instruments

To measure WLB organizational culture, we used 22 questions, which were partially modified and supplemented to meet the purpose of this study, based on Park and Sohn’s [26] five-factor WLB organizational culture scale. A boss’s consideration for WLB means that the boss values WLB, communicates with subordinates, and supports their WLB. A company’s willingness for WLB is a measure of how much the company values WLB and how willing it is to support WLB. The ease of using WLB programs refers to the degree of support needed to easily use programs and systems that support the life of employees. The material support of colleagues for WLB refers to the specific and material support provided by colleagues for WLB. Empathetic communication with colleagues refers to the extent to which one communicates with their colleagues about difficulties related to their WLB. 

To measure WLB, we used four questions based on Kaya and Karatepe [59], which analyzed the effects of WLB on hotel employees in Turkey. Among the four measurement questions, “I have difficulty balancing my work and non-work activities” was reverse-coded as an inverse-scored question. The turnover intention was measured by five questions based on Vanderpool and Way [60], which investigated the chain of relationships between work–family balance, job anxiety, and turnover intention. Each item in the instrument was measured on a five-point Likert type scale with 1 = strongly disagree and 5 = strongly agree.

### 3.3. Data Collection

To verify the hypotheses of the study, a survey was conducted among culinary staff at hotels in Incheon. Owing to its close proximity to Seoul, the capital of Korea, and its international airport, Incheon possess a significant number of hotels to accommodate Korean and foreign visitors. Incheon was chosen for this study because the WLB organizational culture of hotels in other regions, particularly in Seoul, may differ in size of hotel as well as regional characteristics and can offset the organizational culture of hotels in Incheon. Additionally, since January 2015, Korea has been assigning star ratings (1 to 5 stars) to hotels based on four indicators. Specifically, we surveyed four-star and five-star hotels, which are defined as those that offer room service for more than 12 h and have two or more restaurants. Incheon has seven five-star hotels and five four-star hotels [61]. Out of these 12 hotels, staff members of 9 that allowed the head of their culinary department to be surveyed for 20 days from August 10 to 30, 2020 were surveyed. First, we explained the purpose of this study to the culinary staff. After obtaining their consent to participate in the survey, a self-administered paper questionnaire was distributed. Of the 320 questionnaires distributed, 306 were collected, and a total of 290 were used for our empirical analysis, excluding 16 questionnaires that were not completed.

### 3.4. Analysis Method

We analyzed the data using the SPSS 20.0 statistical package program and AMOS 18.0 after the data cleaning and coding process. First, we performed frequency analysis to identify the general and demographic characteristics of the respondents. Second, to verify the validity and reliability of the constituent factors, confirmatory factor analysis and reliability analyses were conducted. Third, we performed correlation analysis to verify the correlation of each variable. Finally, structural equation modeling was applied to evaluate the validity of the proposed model and to verify the hypotheses.

## 4. Results

### 4.1. Participant Characteristics

Table 1 presents the results of the frequency analysis of the demographic characteristics of the respondents. There were 154 males (53.1%) and 136 females (46.9%) in the sample. The number of respondents in their 30s was the highest with 120 (41.4%), which was followed by 86 (29.7%) respondents in their 20s and 64 (22.0%) respondents in their 40s. Out of all the respondents, 161 (55.0%) were married, while 129 (44.5%) were single. In terms of education level, 141 (48.6%) graduated from junior colleges, followed by 97 (33.4%) who graduated from universities. There were 84 (29.0%) respondents at the senior staff level, followed by 74 (25.5%) at the assistant manager level.

### 4.2. Measurement Model

Table 2 shows the results of the confirmatory factor analysis conducted to examine the reliability and validity of each construct constituting the research model. In the analysis, the standardized factor loading value for “My colleagues try to help me with my work when I have family problems”, a measurement item of material support of colleagues for WLB factor, was 0.394, which was below the standard value (0.5); thus, it was eliminated. According to the results of the confirmatory factor analysis, after eliminating this item, the goodness of fit index was χ^2^ = 633.644 (df = 354, *p* < 0.01), χ^2^/df = 1.790, GFI = 0.897, TLI = 0.941, CFI = 0.949, RMSEA = 0.045, and RMR = 0.044. It is verified that this goodness of fit index satisfies the criteria suggested by Hair et al. [62]. Additionally, the Cronbach’s α values were 0.783 or higher for all the seven factors, confirming the reliability of the internal consistencies of the measurement items [63].

Additionally, the validity of the confirmatory factor analysis can be evaluated with convergent and discriminant validity. It is necessary to check whether the standardized factor loading of the measurement items is 0.5 or higher, the composite reliability (CR) is 0.7 or higher, and the average variance extracted (AVE) is 0.5 or higher in order to verify convergent validity [62,64]. According to the convergent validity analysis, the standardized factor loading of WLB organizational culture, the exogenous variable, was between 0.663 and 0.972, and that of WLB and turnover intention was 0.850–0.978, both exceeding the standard value of 0.5. CR and AVE also exceeded their respective standard values, indicating that the analysis is valid.

To verify discriminant validity, it is necessary to check whether the AVE between two constructs is greater than the squared correlation coefficient between them [63]. Table 3 indicates that the squared correlation coefficient (0.41) between the ease of using WLB programs and boss’s consideration for WLB (the highest correlation coefficient value) was lower than the lowest AVE value, which is for the factor of empathetic communication with colleagues (0.639), thereby confirming the discriminant validity of the constructs.

### 4.3. Correlation Analysis

Table 3 shows the results of the correlation analysis conducted before testing the hypotheses for each factor. 

Based on the regression analysis results, turnover intention was negatively correlated with boss’s consideration for WLB (r = −0.623), company’s willingness for WLB (r = −0.568), ease of using WLB programs (r = −0.579), material support of colleagues for WLB (r = −0.569), empathetic communication with colleagues (r = −0.529), and WLB (r = −0.624). There was no factor with a correlation coefficient of 0.8, confirming that there was no problem of multicollinearity.

### 4.4. Structural Equation Modeling

**Figure 2:**
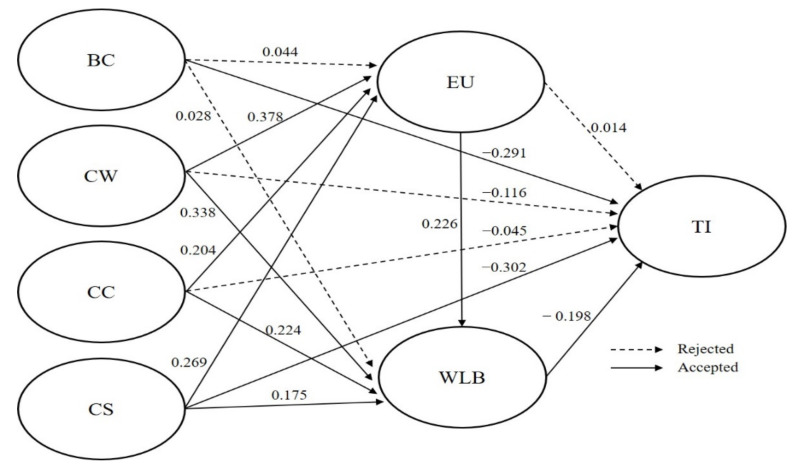
Result of structural equation modeling.

A structural equation model was used to verify the hypotheses of this study. The analysis results are shown in Table 4 and Figure 2. The robustness of the model is Chi-square = 763.949 (df = 384; *p* < 0.001), Chi-square/df = 1.989, NFI = 0.924, TLI = 0.955, CFI = 0.960, RMSEA = 0.059, and SRMR = 0.047. Table 4 shows the results of the significance test on the relationship between variables; the fit indexes satisfied the respective common acceptance levels suggested by Hair et al. [62].

First, boss’s consideration for WLB (BC; β = 0.378, *p* < 0.001), empathetic communication with colleagues (CC; β = 0.204, *p* < 0.001), and material support of colleagues for WLB (CS; β = 0.269, *p* < 0.001) had a positive impact on ease of using WLB programs (EU). These findings support H2, H3, and H4. However, H1 was rejected, since a company’s willingness for WLB (CW; β = 0.044, *p* > 0.05) had no significant impact on EU.

Second, CW (β = 0.338, *p* < 0.001), EU (β = 0.226, *p* < 0.001), CC (β = 0.224, *p* < 0.001), and CS (β = 0.175, *p* < 0.01) were all positively associated with the WLB of culinary staff. These findings support H5b, H5c, H5d, and H5e. However, H5a was rejected, since BC (β = 0.028, *p* > 0.05) had no significant impact on WLB. 

Third, BC (β = −0.291, *p* < 0.001) and CS (β = −0.302, *p* < 0.001) had a negative impact on the turnover intention (TI) of culinary staff. These findings support H6a and H6e. However, CW (β = −0.116, *p* > 0.05), EU (β = 0.014, *p* > 0.05), and CC (β = −0.045, *p* > 0.05) did not have a significant impact on TI. Therefore, H6b, H6c, and H6d were rejected. 

Fourth, WLB (β = −0.198, *p* < 0.01) had a negative impact on the TI of culinary staff. These findings support H7.

Additionally, the structural model had two parameters (WLB and EU), and it was converted using the phantom variables to analyze the specific indirect effect by path, and the indirect effect was verified using AMOS bootstrapping (2000 times) [65]. The results are shown in Table 5. 

First, the EU appears to have an indirect effect on the relationship between BC (B = 0.085, *p* < 0.05), CS (B = 0.06, *p* < 0.01), CC (B = 0.051, *p* < 0.01), and WLB among the four factors of WLB organizational culture. However, EU had no indirect effect on the relationship between CW and WLB (B = 0.01, *p* > 0.05).

Second, the indirect effect of EU was not statistically significant in the relationship between the four factors of WLB organizational culture and TI.

Third, WLB was found to have an indirect effect on the relationship between CW (B = −0.109, *p* < 0.01), CS (B = −0.056, *p* < 0.05), CC (B = −0.081, *p* < 0.01), EU (B = −0.096, *p* < 0.01), and TI. However, WLB had no indirect effect on the relationship between BC and TI (B = −0.009, *p* > 0.01). 

Fourth, on examining the dual mediation effect of EU and WLB in the relationship between the four factors of WLB organizational culture and TI, we found that two parameters had a dual mediation effect in the relationship between BC (B = −0.028, *p* < 0.05), CS (B = −0.02, *p* < 0.05), CC (B = −0.017, *p* < 0.05), and TI. However, there was no dual mediation effect in the relationship between CW and TI (B = −0.003, *p* > 0.05).

## 5. Discussion

This study examined the influence of WLB organizational culture of hotels on WLB and turnover intention of culinary staff. We conducted a survey among culinary staff working at hotels in Incheon, and responses to 290 survey forms were empirically analyzed. 

The results of the study can be summarized as follows: First, it was found that the higher the BC, CS, and CC, the easier it is for culinary staff to use WLB programs. However, even when CW was high, EU did not increase. These results are consistent with the findings of Lee et al. [36] in that support from bosses and colleagues has a positive impact on the ease of using WLB programs, as well as with those of Lee and Lee [38] in that creating a family-friendly work environment may not promote Korean employees’ leave use.

Second, CW, EU, CS, and CC increased the WLB of culinary staff, but not BC. These results are partially consistent with those of Na [34] and Lee and Choi [2].

Third, BC and CS lowered the TI of culinary staff. These results are partially consistent with the results of Na and Kim [51] and Kim and Kim [52] who reported that a sense of support for colleagues and gossip about colleagues affect TI, as well as those of Kim and Chung [50] and Lee et al. [49] who reported that impersonal supervision of bosses and empathy of bosses affect TI. However, EU failed to lower TI. This result is the opposite of the results of Kim et al. [54], who reported that the recognition and ease of using family-friendly programs lower TI.

Fourth, it was found that WLB lowered the TI of culinary staff. These results support the results of Kang et al. [58] and Hong et al. [56] who reported that the higher the WLB of employees working in the hospitality industry, the lower their turnover intention. 

### 5.1. Academic and Practical Implications 

In light of the results of our analysis, the following are the academic and practical implications: First, prior studies in the hospitality industry did not identify what was necessary to improve the WLB of hospitality employees by analyzing the organizational culture of companies and focusing on its hierarchy, development, and rational culture. In this study, measurement tools were used to derive implications for increasing the WLB of hospitality industry workers by understanding the importance of support from bosses, colleagues, and companies. Second, previous studies on organizational culture for WLB had limitations in that they simply analyzed the influence relationship between organizational culture components and employee attitude variables, such as job satisfaction and turnover intention. However, this study has academic significance because EU among the five factors constituting organizational culture was used as a dependent variable, and the research model was expanded by analyzing the relationship between this variable and the remaining four variables.

From a practical implication viewpoint, first, it was found that even with a high CW, hotel chefs were unable to easily use the WLB program, and a high CW did not lower their turnover intention. Hotel chefs are reluctant to use the system owing to fears of retaliation, such as resignations, salary freezes or cuts, and low HR evaluation scores when using WLB-related programs such as paternity leave or maternity leave [53]. Additionally, since the culinary department of a hotel frequently experiences food orders, production, and sales simultaneously, chefs have relatively higher stress compared to those in other departments in the hotel, and interdependent work among co-workers is essential, which indicates that strong cooperation and teamwork among employees are essential. Accordingly, even if the hotel encourages its employees to use the program, chefs do not use it owing to their relationship with work-related colleagues and psychological responsibility. Therefore, hotel management should establish ancillary programs, such as a vacation carry-over system, financial incentives for vacation use (vacation expense support), and penalties for not taking vacation to promote the use of WLB programs under the condition that it does not interfere with their work. However, considering the reality of long working hours in hotel kitchens, it appears more desirable to provide financial incentives rather than granting negative penalties to chefs.

Second, BC did not affect WLB, but communication with colleagues or support from colleagues was found to lower turnover intention through WLB. A vertical organizational structure dominates the hotel cooking department, and therefore, there are many conflict issues between superiors and subordinates; thus, the WLB of culinary staff cannot be improved only by the leadership of seniors. Therefore, hotel management should develop and support programs that would enhance human relationships among members, such as club events and sports activities. To accomplish this, it is necessary to create a sustainable organizational culture where colleagues can understand and support each other when they encounter problems other than those that may occur while balancing work and family. Additionally, the head of the culinary department will need to avoid conducting personnel management according to past practices or the top–down management structure and understand the characteristics of each generation of cooking staff and communicate constantly. Therefore, hotel management must educate and train culinary leaders to recognize the importance of caring for and supporting their staff.

Third, it was found that EU had a positive impact on WLB but failed to reduce the TI of culinary staff unlike employees of other jobs. In other words, no matter how well established a hotel company’s welfare system is, if there is a mismatch between the hiring conditions in the labor market and the job search conditions, employees will leave the organization. In this regard, hotel management should resolve mismatches by identifying the factors that cause chefs to be dissatisfied with their jobs (e.g., wages, working hours) or working values. In particular, to prevent new generation chefs, who value earned income or working hours, from quitting, hotel management needs to identify their reservation wage and provide more financial incentives than other companies, present the potential for personal development, and share corporate vision.

### 5.2. Research Limitation and Future Research

Despite these findings, this study has the following limitations. First, it analyzed how WLB organizational culture affects the WLB of culinary staff. However, a wide range of factors influence the WLB of culinary staff. Hence, it is necessary to expand the research in the future by including diverse factors such as the sex, generation, and work values of culinary staff. Furthermore, this study focused on the culinary staff working at hotels in Incheon. Despite the same rating, the organizational culture of the hotels located in Incheon is different from the WLB of the hotels located in Seoul or other locations. Therefore, future studies can provide more useful implications if a comparative analysis is conducted considering the location, size, or rating of hotels.

## 6. Conclusions

This study analyzed the effect of WLB organizational culture to lower the turnover intention of hospitality employees using two parameters such as EU and WLB. As a result of the analysis, BC, CS, and CC enhanced the EU. However, EU increased the WLB but failed to lower TI. These results mean that the turnover of hospitality industry employees is affected by various factors. Therefore, hotel management should create an organizational culture that supports the WLB of culinary staff. In addition, it is necessary to lower their turnover rate by understanding their job values by generation.

## Figures and Tables

**Table 1:** Participant characteristics.

Characteristics		Frequency	Percentage
Sex	Male	154	53.1
	Female	136	46.9
Age	20s	86	29.7
	30s	120	41.4
	40s	64	22
	50 and above	20	6.9
Marital status	Married	129	44.5
	Single	161	55
Education level	Graduation from high school	27	9.4
	Graduation from junior college	141	48.6
	Graduation from university (four-year)	97	33.4
	Graduation from graduate school	25	8.6
Position	Staff	87	30.1
	Senior staff	84	29
	Assistant manager	74	25.5
	Manager	45	15.5
Total		290	100

**Table 2:** Results of the confirmatory factor analysis and reliability analysis.

Construct		Factor Loading	*t*-Value	AVE	CR
Boss’s consideration for WLB (Cronbach’s α = 0.915)					
bc1	My boss listens attentively to what subordinates have to say about their personal problems.	0.843	Fixed	0.683	0.915
bc2	My boss sympathizes with the difficulties in balancing work and family (child care, parenting, marital problems, etc.).	0.809	16.656 ***		
bc3	My boss is understanding and considerate from the point of view of his/her subordinates.	0.893	19.516 ***		
bc4	My boss values the family life of his/her subordinates	0.786	15.928 ***		
bc5	My boss freely discusses issues related to individual growth (career advancement, promotion, and education) with his/her subordinates.	0.796	16.233 ***		
Company’s willingness for WLB (Cronbach’s α = 0.916)					
cw1	Our hotel strives to provide an environment where the cooks can concentrate on their work without worrying about their family problems.	0.825	Fixed	0.688	0.917
cw2	Our hotel supports various areas of the cook’s life (family, leisure, self-development, etc.).	0.847	17.190 ***		
cw3	Our hotel regards the cook’s leisure time as important.	0.861	17.635 ***		
cw4	Our hotel prioritizes the growth of our hotel and cook together.	0.796	15.699 ***		
cw5	Our hotel values the cook’s WLB.	0.818	16.352 ***		
Ease of using WLB programs (Cronbach’s α = 0.907)					
eu1	Our department allows the chef to be absent or to take an early leave owing to family problems.	0.759	Fixed	0.722	0.911
eu2	Our department does not have a problem with employees taking leaves for personal or family events.	0.956	17.913 ***		
eu3	Our department allows the chef to use work–family support programs (paternity leave, maternity leave, etc.) supported by the company.	0.729	13.024 ***		
eu4	Our department does not have to guess what our boss or colleagues are thinking when taking vacations (annual leave, summer vacation, etc.).	0.930	17.428 ***		
Material support of colleagues for WLB (Cronbach’s α = 0.783)					
cs2	My colleagues help me when I have difficulties (child care, parenting, marital problems, etc.) in balancing work and family.	0.876	Fixed	0.870	0.953
cs3	My colleagues help me when I have personal problems (family, leisure, growth, and self-development).	0.948	25.647 ***		
cs4	My colleagues adjust my working hours when I have personal problems (family, leisure, growth, and self-development).	0.972	27.014 ***		
Empathetic communication with colleagues (Cronbach’s α = 0.870)					
cc1	My colleagues are available to discuss problems related to my personal life (child care, parenting, marital problems, etc.).	0.905	Fixed	0.639	0.874
cc2	My colleagues are attentive to my concerns.	0.740	15.244 ***		
cc3	My colleagues understand the difficulties I have in balancing my work and family (child care, parenting, marital problems, etc.).	0.663	12.943 ***		
cc4	My colleagues are people with whom I can discuss my personal life (family, leisure, growth, and self-development).	0.865	19.706 ***		
WLB perception (Cronbach’s α = 0.925)					
wlb1	I currently have a good balance between the time I spend at work and the time I have for non-work activities.	0.898	Fixed	0.755	0.925
wlb2	There seems to be a healthy balance between my work demands and non-work activities.	0.860	20.777 ***		
wlb3	Overall, I believe that my work and non-work life are balanced.	0.850	20.290 ***		
wlb4	I have difficulty balancing my work and non-work activities.	0.868	21.195 ***		
Turnover intention (Cronbach’s α = 0.989)					
ti1	There is a high probability that I will actively seek employment with a different organization in the next year.	0.966	Fixed	0.947	0.989
ti2	I have seriously considered changing organizations since I began working here.	0.979	49.664 ***		
ti3	I will not be working here after a year.	0.973	47.154 ***		
ti4	I do not intend to remain with this hotel for more than a few years.	0.978	49.599 ***		
ti5	Currently, I am actively searching for another job in a different organization.	0.970	46.053 ***		

Note: BC = boss’s consideration for WLB; CW = company’s willingness for WLB; EU = ease of using WLB programs; CS = material support of colleagues for WLB; CC = empathetic communication with colleagues; WLB = work–life balance; TI = turnover intention; AVE = average variance extracted; CR = composite reliability; Chi-square = 763.949 (df = 384), *p* < 0.000, Chi-square/df = 1.989; normed fit index (NFI) = 0.924, relative fit index (RFI) = 0.914, incremental fit index (IFI) = 0.961, Tucker–Lewis index (TLI) = 0.955, comparative fit index (CFI) = 0.960, root square error of approximation (RMSEA) = 0.059, standardized root mean square residual (SRMR) = 0.047; *** *p* < 0.001.

**Table 3:** Correlation analysis and discriminant validity test.

Construct	Mean ± S.D.	BC	CW	EU	CS	CC	WLB	TI
BC	3.36 ± 0.88	0.683 ^(1)^	0.298 ^(3)^	0.412	0.261	0.272	0.309	0.388
CW	2.86 ± 0.96	0.546 *** ^(2)^	0.688 ^(1)^	0.250	0.225	0.233	0.393	0.323
EU	3.34 ± 0.78	0.642 ***	0.500 ***	0.722 ^(1)^	0.361	0.329	0.404	0.335
CS	3.41 ± 0.99	0.511 ***	0.474 ***	0.601 ***	0.870 ^(1)^	0.222	0.327	0.408
CC	3.08 ± 0.73	0.522 ***	0.483 ***	0.574 ***	0.471 ***	0.639 ^(1)^	0.343	0.280
WLB	3.08 ± 0.87	0.556 ***	0.627 ***	0.636 ***	0.572 ***	0.586 ***	0.755 ^(1)^	0.389
TI	2.71 ± 1.49	−0.623 ***	−0.568 ***	−0.579 ***	−0.639 ***	−0.529 ***	−0.624 ***	0.947 ^(1)^

Note: ^(1)^ Diagonal values show AVE; ^(2)^ The values in the lower left off-diagonal show the correlation coefficient; ^(3)^ The values in the upper right off-diagonal show the squared correlation coefficient; S.D. = standard deviation; TI = turnover intention; *** *p* < 0.001.

**Table 4:** Results of structural equation modeling.

Hypothesized Path				Estimate		S.E.	*t*-Value	Results
				B	Beta			
H1	CW	→	EU	0.033	0.044	0.046	0.731	Rejected
H2	BC	→	EU	0.288	0.378	0.053	5.482 ***	Accepted
H3	CC	→	EU	0.174	0.204	0.053	3.300 ***	Accepted
H4	CS	→	EU	0.204	0.269	0.044	4.641 ***	Accepted
H5a	BC	→	WLB	0.027	0.028	0.066	0.414	Rejected
H5b	CW	→	WLB	0.332	0.338	0.058	5.714 ***	Accepted
H5c	EU	→	WLB	0.293	0.226	0.085	3.447 ***	Accepted
H5d	CC	→	WLB	0.249	0.224	0.066	3.768 ***	Accepted
H5e	CS	→	WLB	0.172	0.175	0.055	3.144 **	Accepted
H6a	BC	→	TI	−0.476	−0.291	0.106	−4.469 ***	Accepted
H6b	CW	→	TI	−0.188	−0.116	0.099	−1.902	Rejected
H6c	EU	→	TI	0.031	0.014	0.137	0.223	Rejected
H6d	CC	→	TI	−0.082	−0.045	0.109	−0.757	Rejected
H6e	CS	→	TI	−0.492	−0.302	0.090	−5.474 ***	Accepted
H7	WLB	→	TI	−0.327	−0.198	0.119	−2.746 **	Accepted

Note: S.E. = standard error; Chi-square = 763.949 (df = 384), *p* < 0.000, Chi-square/df = 1.989, NFI = 0.924, RFI = 0.914, IFI = 0.961, TLI = 0.955, CFI = 0.960, RMSEA = 0.059, SRMR = 0.047; ** *p* < 0.01, *** *p* < 0.001.

**Table 5:** Results of the mediation effect.

Path	B	S.E.	Beta
BC→TI	−0.476 ***	0.106	−0.291
BC→EU→WLB	0.085 *	0.03	0.085
BC→EU→TI	0.009	0.044	0.005
BC→WLB→TI	−0.009	0.024	−0.006
BC→EU→WLB→TI	−0.028 **	0.016	−0.017
CW→TI	−0.188	0.099	−0.116
CW→EU→WLB	0.010	0.015	0.010
CW→EU→TI	0.001	0.009	0.001
CW→WLB→TI	−0.109 **	0.048	−0.067
CW→EU→WLB→TI	−0.003	0.006	−0.002
CS→TI	−0.492 ***	0.09	−0.302
CS→EU→WLB	0.060 **	0.023	0.061
CS→EU→TI	0.006	0.032	0.004
CS→WLB→TI	−0.056 *	0.030	−0.035
CS→EU→WLB→TI	−0.02 *	0.011	−0.012
CC→TI	−0.082	0.109	−0.045
CC→EU→WLB	0.051 **	0.022	0.046
CC→EU→TI	0.005	0.028	0.003
CC→WLB→TI	−0.081 **	0.042	−0.044
CC→EU→WLB→TI	−0.017 *	0.010	−0.009
EU→TI	0.031	0.137	0.014
EU→WLB→TI	−0.096 *	0.051	−0.045

Note: * *p* < 0.05, ** *p* < 0.01, *** *p* < 0.001.

## Data Availability

The data presented in this study are available on request from the corresponding author. The data are not publicly available due to confidentiality agreements with participants.
